# Transcriptome comparison of the sex pheromone glands from two sibling *Helicoverpa* species with opposite sex pheromone components

**DOI:** 10.1038/srep09324

**Published:** 2015-03-20

**Authors:** Zhao-Qun Li, Shuai Zhang, Jun-Yu Luo, Chun-Yi Wang, Li-Min Lv, Shuang-Lin Dong, Jin-Jie Cui

**Affiliations:** 1 State Key Laboratory of Cotton Biology, Institute of Cotton Research of CAAS, Anyang 455000, China; 2 College of Plant Protection, Nanjing Agricultural University/Key Laboratory of Integrated Management of Crop Diseases and Pests (Nanjing Agricultural University), Ministry of Education, Nanjing 210095, China

## Abstract

Differences in sex pheromone component can lead to reproductive isolation. The sibling noctuid species, *Helicoverpa armigera* and *Helicoverpa assulta*, share the same two sex pheromone components, Z9-16:Ald and Z11-16:Ald, but in opposite ratios, providing an typical example of such reproductive isolation. To investigate how the ratios of the pheromone components are differently regulated in the two species, we sequenced cDNA libraries from the pheromone glands of *H. armigera* and *H. assulta*. After assembly and annotation, we identified 108 and 93 transcripts putatively involved in pheromone biosynthesis, transport, and degradation in *H*. *armigera* and *H*. *assulta*, respectively. Semi-quantitative RT-PCR, qRT-PCR, phylogenetic, and mRNA abundance analyses suggested that some of these transcripts involved in the sex pheromone biosynthesis pathways perform. Based on these results, we postulate that the regulation of desaturases, KPSE and LPAQ, might be key factor regulating the opposite component ratios in the two sibling moths. In addition, our study has yielded large-scale sequence information for further studies and can be used to identify potential targets for the bio-control of these species by disrupting their sexual communication.

In insects, species-specific behaviours elicited by sex pheromones play a key role in reproduction and are associated with reproductive isolation^1^. The regulation of sex pheromone-related enzymes lead to speciation by changing mate recognition systems. In moths, most sex pheromones components are C_10_–C_18_ long-chain unsaturated alcohols, aldehydes or acetate esters that are produced *de novo* via a modified fatty-acid biosynthesis pathway in the sex pheromone glands (PGs) by acetylation, desaturation, chain shortening, reduction, and oxidation either separately or in combination^2^,^3^. Different combinations of these reactions produce unique species-specific pheromone blends in different species.

Sex pheromone biosynthesis in moths starts with the production of the saturated fatty-acid precursor, malonyl-CoA, from acetyl-CoA and is catalysed by acetyl-CoA carboxylase (ACC) and fatty acid synthase (FAS)^4^. Then, the fatty chain is modified to introduce a double bond by specific desaturases (DESs), and shorted by β-oxidation^5^. Thus far, six types of DES have been functionally characterized, including Δ5^6^, Δ6^7^, Δ9^8^, Δ11^9^, Δ10–12^10^, and Δ14^11^. After the production and release of the sex pheromone components by females, the pheromone molecules are captured by odorant binding proteins (OBPs)^12^,^13^,^14^ or chemosensory proteins (CSPs)^15^ and transported to membrane-bound olfactory receptors (ORs)^16^,^17^,^18^. After OR activation, the pheromone molecules are rapidly removed by odorant degrading enzymes (ODEs), such as carboxylesterase^19^ and aldehyde oxidases (AOXs)^20^ to restore the sensitivity of the sensory neuron. Analysing these genes involved in the production of specific pheromone components will provide insights into the regulation of the pheromone component and thereby the evolution of moth sexual communication.

The lepidopterans, *Helicoverpa armigera* and *Helicoverpa assulta* are two sympatric sibling species that are morphologically indistinguishable in the egg, larval, and pupal stages^21^. Furthermore, these two species share the common sex pheromone components, Z9-16:Ald and Z11-16:Ald, but the ratios between the two components is completely reversed^22^,^23^, 100:7 in *H. armigera* and 7:100 in *H. assulta*. It is plausible that this difference likely contributes to the reproductive isolation of the two species. Some studies have been carried out to explore the regulatory mechanisms that determine these species-specific ratios^22^, but the mechanisms remains not well known especially from the molecular perspective. Therefore, we constructed and sequenced cDNA libraries from the PGs isolated from *H. armigera* and *H. assulta* to investigate the genetic factors associated with sex pheromone biosynthesis in these two species.

After analysis, we identified 108 and 93 putative pheromone biosynthesis, transport, and degradation transcripts in the PGs of *H. armigera* and *H. assulta*, respectively. Our results together with previous studies^22^,^24^ support the conjecture that the regulation of DESs is likely to play an important role in determining the opposite sex pheromone components ratios in the two species. In addition, our results also provide large-scale sequence information for further studies and identification of potential targets to disrupt sexual communication in *H. armigera* and *H. assulta* for the control of these lepidopterans.

## Results

### Overview of the PG transcriptomes

**Figure 1:**
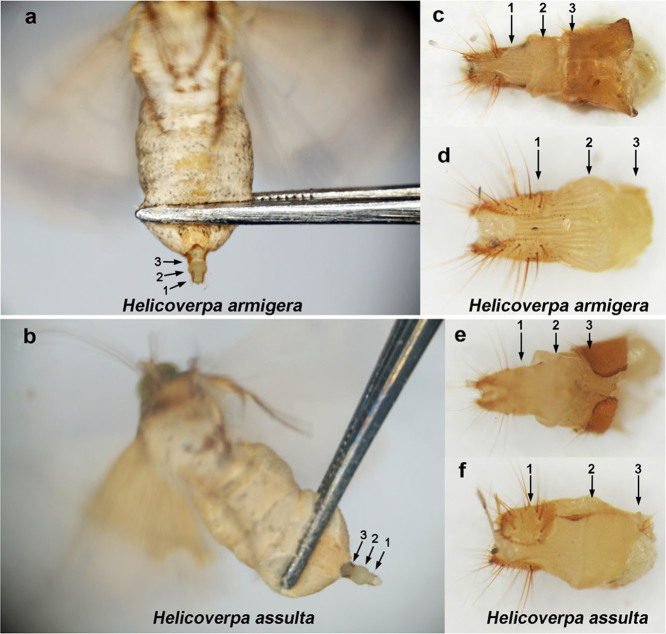
Dissection of *Helicoverpa armigera* and *Helicoverpa assulta* sex pheromone glands. The pheromone glands in *H. armigera* (a) and *H. assulta* (b) were squeezed out from the abdomen using forceps (the gland is similarly inflated when the female calls). The abdomen of *H. armigera* (c) and *H. assulta* (e) were cut at the sclerotized cuticle from the 8^th^ abdominal segment, and the sclerotized cuticle was removed (*H. armigera* (d) and *H. assulta* (f)) before immersing the glands in liquid nitrogen. **1:** Sclerotized ovipositor valves; **2:** Pheromone gland; **3:** Sclerotized cuticle that was removed.

PGs from *H. armigera* and *H. assulta* were collected as previously described for the *Heliothis virescens* PG transcriptome^25^ (Fig. 1) followed by construction of the corresponding cDNA libraries. Large-scale transcripts were assembled and annotated in the PG transcriptomes from *H. armigera* and *H. assulta* (Supplementary Table S1 online).

**Figure 2:**
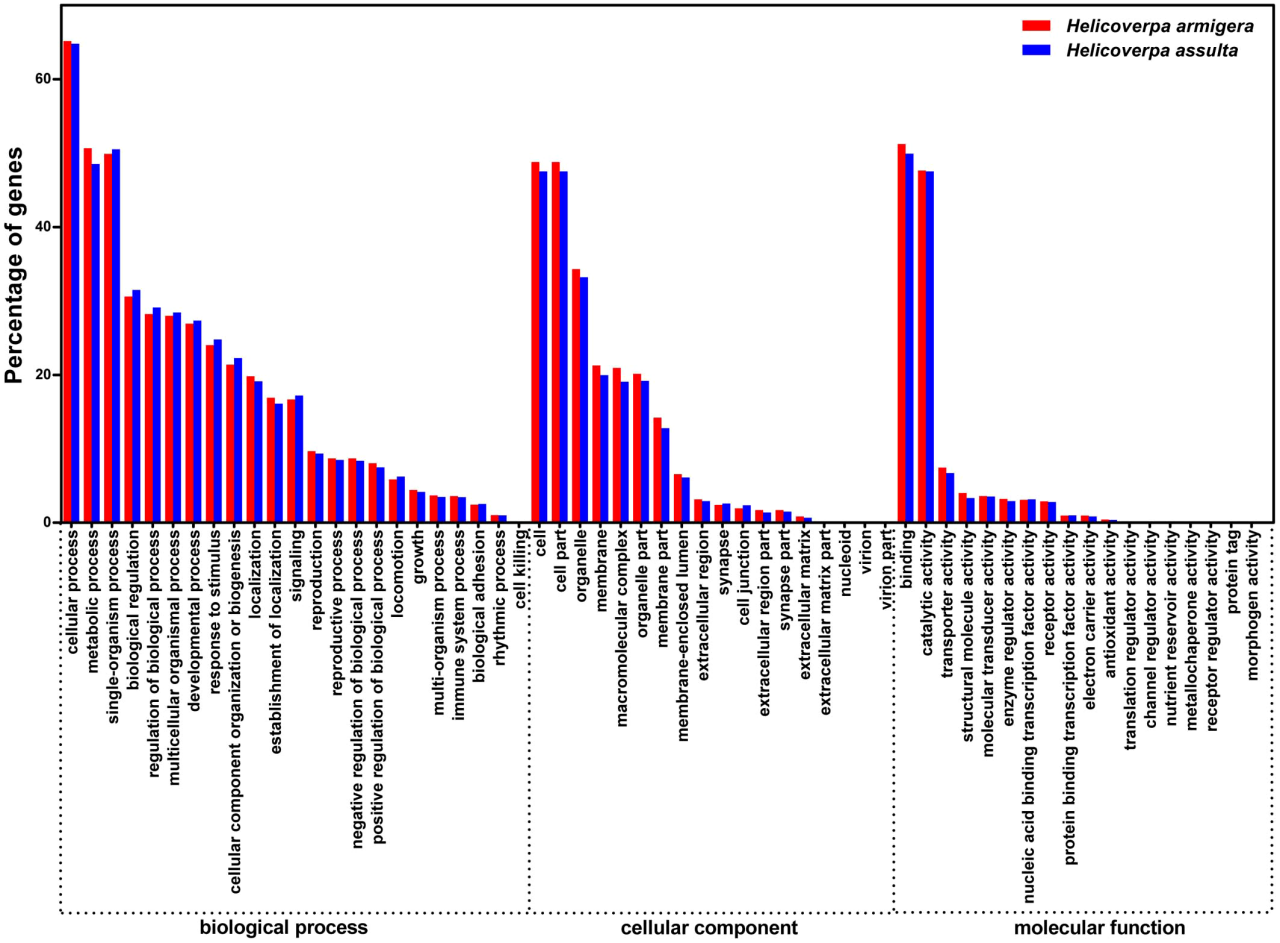
Distribution of transcripts in *Helicoverpa armigera* and *Helicoverpa assulta* pheromone glands. All transcripts were annotated using Gene Ontology and their distribution in the three major GO categories is shown. The analysis was at level 3.

GO annotation was used to classify the PG transcripts into functional categories. GO terms were represented in all three major GO categories: biological process, cellular component, and molecular function. The most represented sub-category in the biological process category was cellular process, in the cellular component category it was cell and cell part, and in the molecular functions category, binding and catalytic activity were the most represented (Fig. 2).

### Identification of putative genes involved in pheromone biosynthesis, transport, and degradation in the two *Helicoverpa* species

After removal of repetitive sequences following blastX against the NCBI Nr database and alignment with ClustalX 2.0, we identified a total of 108 and 93 putative transcripts involved in the pheromone biosynthesis, transport, and degradation in *H. armigera* and *H. assulta* PGs, respectively (Tables 1 and 2). These transcripts belonged to gene families represented by multiple transcripts in these two moth species. For example, *ACC* had 2 members in the 2 species each, *alcohol dehydrogenase* (*ALR*) was represented by 17 and 18 sequences in *H. armigera* and *H. assulta*, *DES* with 7 and 8, *FAS* with 3 and 3, *FAR* with 18 and 13, *CSP* with 19 and 16, *OBP* with 26 and 23, *aldehyde dehydrogenase* (*AD*) with 9 and 6, and *AOX* with 7 and 4 members respectively, in *H. armigera* and *H. assulta* (Tables 1 and 2, Supplementary Tables S2–S5 online).

### Tissue expression profile and mRNA abundance of the sex pheromone biosynthesis putative genes

**Figure 3:**
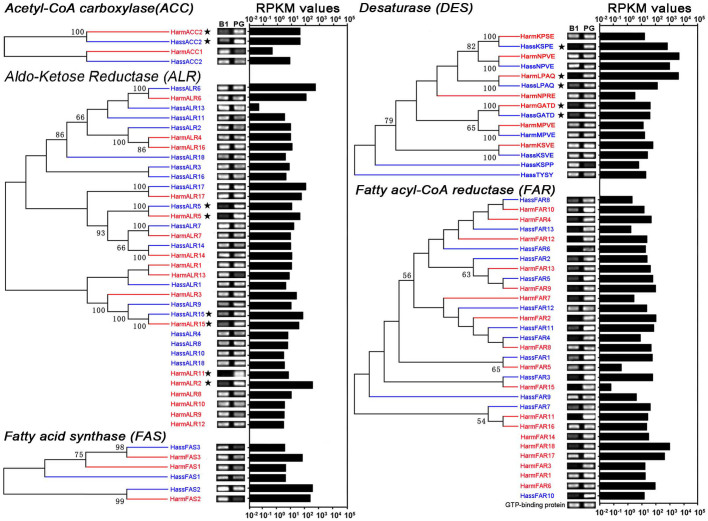
Phylogenetic analysis, expression profiles and abundances of pheromone biosynthesis-related transcripts in *Helicoverpa armigera* and *Helicoverpa assulta*. The phylogenetic tree was constructed in MEGA6.0 using the neighbour-joining method. Bootstrap values >50% (1000 replicates) are indicated at the nodes. Transcripts that were too short for phylogenetic analysis are listed under the respective trees. Expression levels of acetyl-CoA carboxylase, aldo-Ketose Reductase, desaturase and fatty acyl-CoA related transcripts were determined in female bodies without pheromone glands (B1) and PGs by semi-quantitative RT-PCR. Transcripts from *H. armigera* are labelled in red and *H. assulta* in blue. Transcript expression abundance is indicated as RPKM values. The PG-biased *ACC*, *ALR*, *FAS,* and *DES*s are labelled with pentagrams in the phylogenetic tree. The gene for GTP-binding protein was used as the positive control.

**Figure 4:**
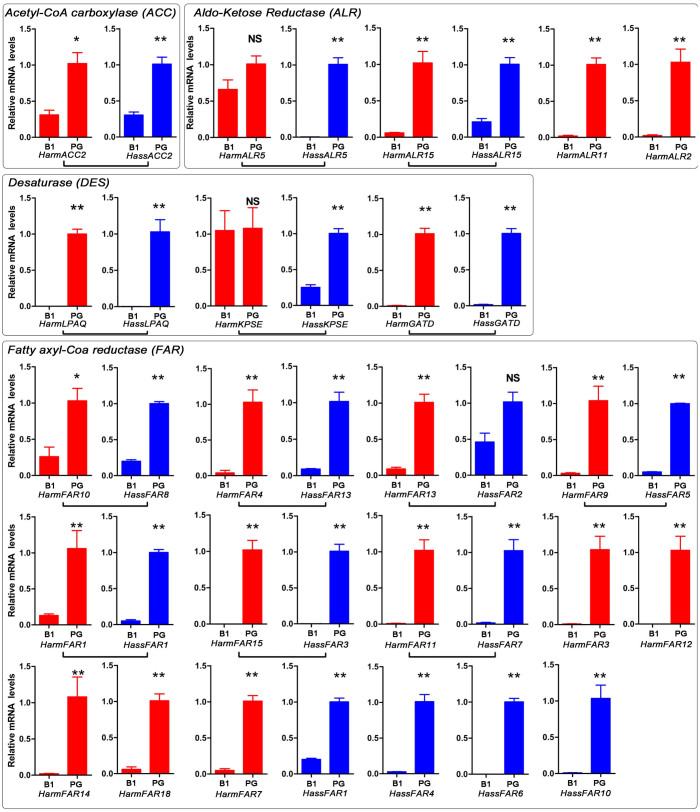
Relative expression levels of *Helicoverpa armigera* and *Helicoverpa assulta* transcripts with PG-biased expression in different female tissues. Expression levels of acetyl-CoA carboxylase, aldo-Ketose Reductase, desaturase and fatty acyl-CoA related transcripts were determined in female bodies without pheromone glands (B1) and PGs by qRT-PCR. Transcripts from *H. armigera* are labelled in red and *H. assulta* in blue. An asterisk indicates a significant difference between the expression levels in female body and PGs (*P* < 0.05, Student's *t*-test). “NS” indicates no significant difference (*P* > 0.05).

We further characterized the expression levels and tissue expression pattern of the transcripts putatively involved in pheromone biosynthesis by semi-quantitative RT-PCR and qRT-PCR. Transcript abundance in the PG was also calculated as RPKM (reads per kilobase per million mapped reads). For this analysis, *H. armigera* sequences had the prefix *Harm* and *H. assulta* sequences had the prefix *Hass* followed by the gene name. The results showed that all the analysed transcripts had different expression patterns and most orthologous transcripts had similar expression profiles (Figs. 3 and 4).

We identified two *ACCs* from the PGs of both *H. armigera* and *H. assulta* (Tables 1 and 2). Semi-quantitative RT-PCR and qRT-PCR results revealed that *HarmACC2* and *HassACC2* were highly expressed in PGs compared to the female body without the PGs (Figs. 3 and 4). Their transcript abundance was also markedly higher (40.9 and 41.5 RPKM) than *HarmACC1* and *HassACC1* (0.4 and 7.7 RPKM) in the transcriptomes.

Three *FASs* were identified in the PGs from *H. armigera* and *H. assulta* (Tables 1 and 2). Semi-quantitative RT-PCR revealed that all three transcripts were expressed at higher levels in the female body when compared to the PGs (Figs. 3 and 4). However, the RPKM values indicated that both *HarmFAS2* (237.5) and *HassFAS2* (345.7) were abundant in the PG transcriptomes. The RPKM values of *HarmFAS2* and *HassFAS2* were 3- and 82-fold higher than the other transcripts in the PG transcriptomes.

Seven and eight *DES*s were identified in the PGs of *H. armigera* and *H. assulta*, respectively (Tables 1 and 2). Semi-quantitative RT-PCR and qRT-PCR results showed that *HarmLPAQ*, *HarmGATD*, *HassLPAQ*, *HassGATD*, and *HassKPSE* had robust expression in the PGs when compared to the female body (Figs. 3 and 4).

To evaluate transcript expression abundances, the RKPM values of DESs, *HarmKPSE* (16.6 RPKM), *HarmGATD* (41.5 RPKM), *HarmLPAQ* (3975.6 RPKM), *HassKPSE* (659.9 RPKM), *HassGATD* (40.0 RPKM), and *HassLPAQ* (132.8 RPKM), were calculated (Tables 1 and 2, and Fig. 3). In comparison, *HarmLPAQ* (Δ11) was highly abundant in the *H. armigera* PG transcriptome, *HassLPAQ* and *HassKPSE* were highly abundant in the *H. assulta* PG transcriptome. The abundance of *HassKPSE* (Δ9) was 7-fold higher in the *H. assulta* PG transcriptome than *HassLPAQ* (Δ11), and *HarmLPAQ* (Δ11) was 239-fold higher in the *H. armigera* PG transcriptome than *HassKPSE* (Δ9). In addition, the abundance of *HassKPSE* in the *H. assulta* was 39-fold higher than *HarmKPSE* in *H. armigera*, while *HassLAPQ* was 30-fold lower than *HarmLPAQ*. *HarmGATD* and *HassGATD* had lower abundances in PG transcriptomes compared to *HarmLPAQ*, *HassKPSE* and *HassLPAQ*.

There were 18 and 13 *fatty acyl-CoA reductases* (*FAR*) in the *H. armigera* and *H. assulta* PG transcriptomes, respectively (Tables 1 and 2). Among the 18 *FARs* in *H. armigera*, *HarmFAR12* (FKPM = 414.1) was more abundant in the PG transcriptome than the other 11 PG-biased *FARs* (RPKM < 70) (Table 1, and Figs. 3 and 4). In *H. assulta, HassFAR6* (RPKM 960.1) was more abundant in the PG transcriptome than the other PG-biased *FARs* (RPKM < 102) (Table 2, and Figs. 3 and 4).

ALR is involved in converting an alcohol to an aldehyde. We identified 17 and 18 *ALRs* in the *H. armigera* and *H. assulta* PG transcriptomes, respectively (Tables 1 and 2). Semi-quantitative RT-PCR and qRT-PCR results indicated that *HarmALR15*, *HarmALR11*, and *HarmALR2* in *H. armigera*, and *HassALR5* and *HassALR15* in *H. assulta* had PG-biased expression (Figs. 3 and 4). In *H. armigera, HarmALR2* was highly abundant (RPKM = 323.6) in the PG transcriptome than the other PG-biased *ALRs* (RPKM < 35) (Tables 1 and 2, and Fig. 3). In *H. assulta, HassALR15* had a higher RPKM (66.1) value than *HassALR5* (10.7).

### Phylogenetic analyses of the DESs

**Figure 5:**
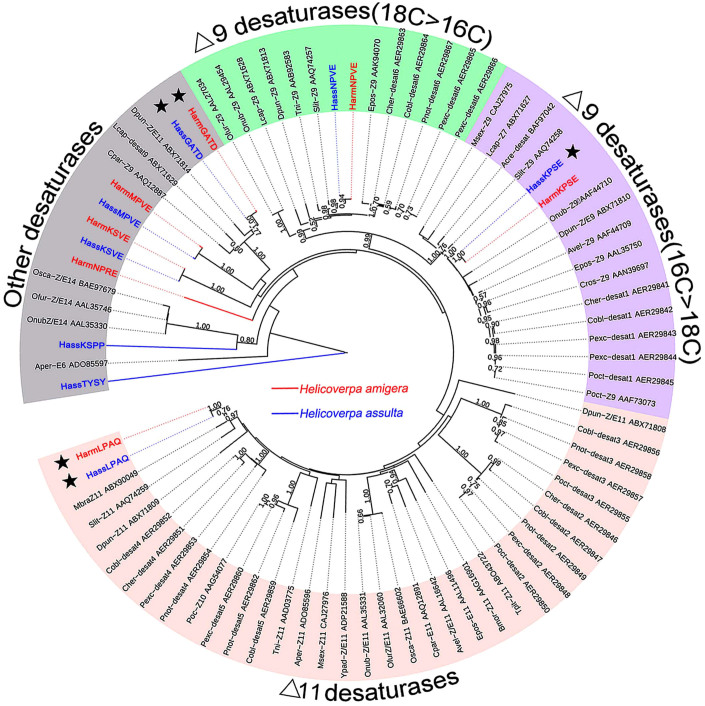
Phylogenetic tree of putative DES from *Helicoverpa armigera* and *Helicoverpa assulta* and other known DESs from lepidopterans. The phylogenetic tree was constructed with MEGA6.0 using the neighbour-joining method. Bootstrap values >50% (1000 replicates) are indicated at the nodes. Transcripts from *H. armigera* are labelled in red and *H. assulta* in blue. DESs with PG-bias are indicated with pentagrams.

To further investigate the function of the DESs from *H. armigera* and *H. assulta*, 15 candidate DESs from these two species were phylogenetically analysed with other lepidopteran DESs (Fig. 5). In the resulting phylogenetic tree, we observed three well-supported clades including Δ9-desaturases (16C > 18C), Δ9-desaturases (16C < 18C), and Δ11-desaturases. The five PG-biased transcripts from *H. armigera* and *H. assulta* were well separated from each other, with many other lepidopteran DESs interspersed among them. HarmLPAQ was very close to HassLPAQ in the Δ11-desaturases clade, and HassKPSE was a member of the Δ9-desaturases (16C > 18C) group. Interestingly, HarmKPSE, did not show PG-biased expression (Figs. 3 and 4) although it was present in the same clade as HassKPSE and the two proteins shared high amino acid identity (99.72%). Similarly, HarmLPAQ and HassLPAQ also shared high amino acid identity (99.70%). It is notable that two transcripts with PG-biased expression, *HarmGATD* and *HassGATD*, did not belong to any of the three main clades.

### Tissue expression profiles of the sex pheromone transport putative genes

**Figure 6:**
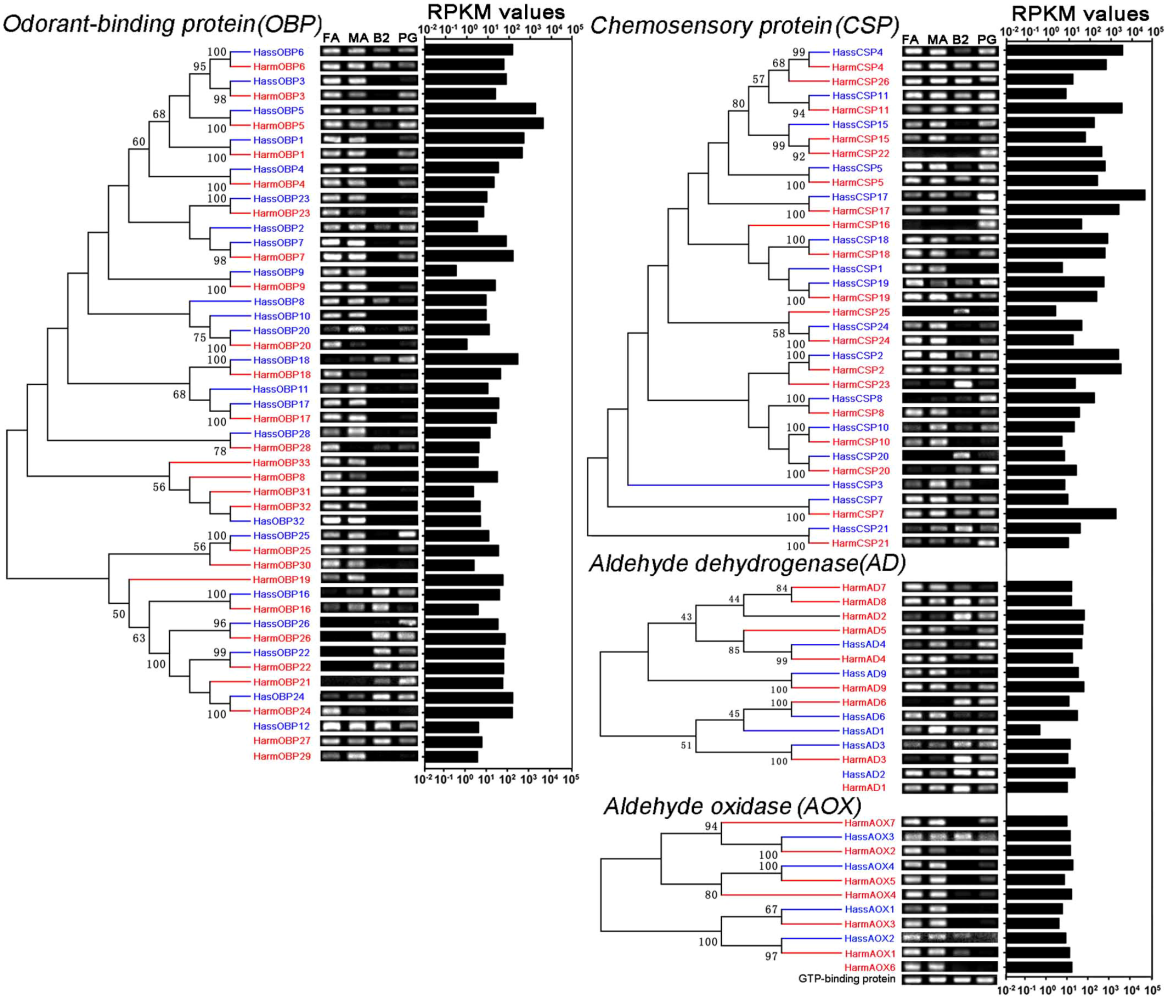
Phylogenetic analysis, expression profiles and abundances of pheromone transport- and degradation-related transcripts in *Helicoverpa armigera* and *Helicoverpa assulta*. The phylogenetic tree was constructed with MEGA6.0 using the neighbour-joining method. Bootstrap values >50% (1000 replicates) are indicated at the nodes. Transcripts that were too short for phylogenetic analysis are listed under the respective phylogenetic trees. Expression levels of odorant-binding proteins, chemosensory proteins, aldehyde dehydrogenase and aldehyde oxidase were determined in female antennae (FA), male antennae (MA), female bodies without pheromone glands and antennae (B2) and PGs by semi-quantitative RT-PCR. Transcripts from *H. armigera* are labelled in red and *H. assulta* in blue. Transcript expression abundance is indicated by RPKM values. The gene for GTP-binding protein was used as the positive control.

We identified 19 and 16 *CSPs,* and 26 and 23 *OBPs* in *H*. *armigera* and *H*. *assulta*, respectively (Supplementary Tables S2 and S3 online). Semi-quantitative RT-PCR results indicated that the orthologous transcripts had similar expression profiles (Fig. 6). Most of the *OBPs* were highly expressed in antennae and/or PGs, indicating their function in the detection and protection of plant volatiles, oviposition-deterring pheromones, and sex pheromones. Most *CSPs* were expressed in a range of tissues, suggesting common functions. Similar to *OBPs*, several *CSP*s in the *Helicoverpa* species were highly expressed in antennae and/or PGs (Fig. 6).

### Tissue expression profiles of sex pheromone degradation putative genes

We identified nine and six *ADs,* and seven and four *AOXs* in *H*. *armigera* and *H*. *assulta*, respectively (Supplementary Tables S4 and S5 online). Semi-quantitative RT-PCR revealed that *HarmAD4*, *HarmAOX7*, *HarmAOX2*, *HarmAOX3*, *HarmAOX4*, *HarmAOX5*, and *HarmAOX6* in *H*. *armigera* and *HassAD9*, *HassAOX3*, and *HassAOX5* in *H*. *assulta* were mainly expressed in antennae and PGs (Fig. 6).

## Discussion

Speciation in insects is often associated with changes in mate recognition systems. Particularly, sex pheromone-induced behaviours play crucial roles in insect reproduction and contribute significantly to reproductive isolation^26^. In moths, sex pheromones are synthesized in the PGs. Both *H. armigera* and *H. assulta*, which are sibling noctuid species, use the sex-pheromone components, Z9-16:Ald and Z11-16:Ald. However, the components are present in opposite ratios in the two species. Intrigued by this, we investigated differences in the transcripts related to sex pheromone biosynthesis, transport and degradation in the two sibling species by sequencing the transcriptomes from the PGs of the two *Helicoverpa* species.

A total of 108 and 93 putative pheromone biosynthesis, transport, and degradation transcripts were respectively identified in *H. armigera* and *H. assulta* PGs. Further characterization of these transcripts by semi-quantitative RT-PCR, qRT-PCR, phylogenetic, and mRNA abundance analyses revealed that some of the transcripts had three characteristics: 1) transcripts that are distinctly or highly expressed in PGs than female body (without PG), 2) transcripts that are more abundant than the other transcripts in the PGs, and 3) transcripts that are homologous to other insect genes with demonstrated function in sex pheromone biosynthesis.

Generally, the pheromone biosynthesis pathway in moths begins with the ATP-dependent carboxylation of acetyl-CoA to malonyl-CoA catalysed by ACC^27^. Compared to other *ACCs*, *HarmACC2* and *HassACC2* were highly expressed in PGs and were highly abundant than the other *ACCs*. In pheromone biosynthesis, FAS has been shown to use malonyl-CoA and NADPH to produce fatty acids^4^. In this study, none of the *FASs* displayed PG-biased expression, although *HarmFAS2* and *HassFAS2* were highly abundant in the PG transcriptomes with high RPKM values compared to other *FASs*. Future studies on the functional characterization of the *Helicoverpa* *ACCs* and *FASs* may reveal their specific roles in pheromone biosynthesis.

During sex pheromone biosynthesis, DESs introduces double bonds at specific positions in fatty acid chains^28^,^29^. Previous studies with labelled fatty acids demonstrated that different pathways are used in the pheromone biosynthesis in *H. armigera* and *H. assulta*^22^ to achieve the markedly different ratios in the sex pheromone components, Z9-16:Ald and Z11-16:Ald. Among the *DESs* identified in our study, *HarmGATD*, *HarmLPAQ*, *HassKPSE*, *HassGATD*, and *HassLPAQ* displayed PG-biased expression compared with the adult female body. However, phylogenetic analyses showed that *HarmGATD* and *HassGATD* were not clustered into groups that were previously demonstrated to function in sex pheromone biosynthesis. On the other hand, HarmLPAQ and HassLPAQ were the members of Δ11-desaturases group, and HarmKPSE was closely related to HassKPSE in the Δ9-desaturases (16C > 18C) group. These two groups of desaturases share a conserved biological function in sex pheromone biosynthesis^30^. Previous studies on desaturases from *H. assulta*^24^,^31^, *Helicoverpa* *zea*^32^ and *Trichoplusia ni*^9^,^33^ showed that *HassKPSE* encodes a Δ9-desaturase. The action of this Δ9-desaturase results in the production of higher amounts of Z9-16:Acid than Z9-18:Acid^24^. *HassLPAQ* shown to encode a Δ11-desaturase that specifically produced Z11-16:Acid. HzPGDs2^32^, TniKPSE^33^ and HassKPSE^24^ have high amino acid identity, sharing the similar function. In addition, the function of HassLPAQ^24^, HzPGDs1^32^ and TniLPAQ^9^ were similar to each other. Considering the amino acid high identity (about 99.7% with only one amino acid difference) between HarmKPSE and HassKPSE, and HarmLPAQ and HassLPAQ, it is likely that HarmKPSE and HassKPSE encode Δ9-desaturases, HarmLPAQ and HassLPAQ encode Δ11-desaturases in *H. armigera* and *H. assulta.*

Interestingly, *HarmKPSE* did not show PG-biased expression, suggesting that this gene is not involved in the sex pheromone biosynthesis. This results is well consistent with the previous labelling study^22^ with D_3_-16:Acid and D_3_-18:Acid showed that Z11-16:Ald is produced by Δ11 desaturation of 16:Acid in both *H. armigera* and *H. assulta*. However, Z9-16:Ald is produced by Δ11 desaturation of 18:Acid and one cycle of two-carbon chain shortening in *H. armigera*, while Z9-16:Ald is mainly produced by Δ9 desaturation of 16:Acid and by Δ11 desaturation of 18:Acid and one cycle of two-carbon chain shortening in *H. assulta*^22^.Therefore, unlike the *HassKPSE*, *HarmKPSE* that encodes a Δ9-desaturase is not likely to be involved in sex pheromone biosynthesis.

On the other hand, PG abundance is another characteristic feature of the genes involved in sex pheromone biosynthesis. The high abundance of *HarmLPAQ*, *HassKPSE* and *HassLPAQ* in the PG transcriptomes suggest that these high abundance and PG biased transcripts may have a role in sex pheromone biosynthesis in the two *Helicoverpa* species. Furthermore, the abundance of *HassKPSE* (Δ9) was 7-fold higher than *HassLPAQ* (Δ11) in the *H. assulta* PG transcriptome was consistent with the major pheromone component being Z9-16:Ald in *H. assulta*. As compared with *HarmLPAQ*, the lower abundance of *HarmKPSE* (about 239-fold) is consistent with that *HarmKPSE* is not likely to be involved in sex pheromone biosynthesis. Together our data along with others reported previously^24^,^22^ suggest that among the *DESs* identified in our study, only *HarmLPAQ* (Δ11) is likely involved in sex pheromone biosynthesis in *H. armigera*, while both *HassLPAQ* (Δ11) and *HassKPSE* (Δ9) may be involved in this process in *H. assulta*.

Mutations that affect gene regulation could be more important in evolution than those changing the amino acid sequence of a protein^34^. In our study, HarmKPSE and HassKPSE had high amino acid identity (99.72%) indicating similar function. But, their expression patterns were different, and the mRNA abundance of *HassKPSE* was 39-fold higher in the *H. assulta* PG than *HarmKPSE* in *H. armigera* PG, while *HassLPAQ* was 30-fold lower than *HarmLPAQ*. Therefore, we presume that the regulation of *DESs* in these two *Helicoverpa* species likely resulted in the evolution of different pathways in the sex pheromone biosynthesis resulting in the final opposite ratios between two sex pheromone components. Further studies on regulation of *DESs* and its function are needed to determine their specific roles in the biosynthesis pathways of these two *Helicoverpa* species.

After the introduction of a specific double bond in the sex pheromone biosynthesis pathway, the fatty acyl CoA pheromone precursors are further reduced to the corresponding alcohols by FAR^35^,^36^,^37^ and then catalysed by ALR. Among the *FARs* and *ALRs* identified in this study, *HarmFAR12*, *HassFAR6*, *HarmALR2*, and *HassALR15* not only showed PG-biased expressions but also displayed a higher abundance than the others in the PGs suggesting their role in sex pheromone biosynthesis.

Some olfactory sensilla are distributed on the ovipositor^38^,^39^, which may function in the olfactory detection of plant odours, ovipositor-deterring pheromones, and sex pheromones. OBPs and CSPs are thought to be responsible for the binding and transport of hydrophobic molecules, including pheromones and plant volatiles^13^,^15^. After sex pheromones have stimulated the olfactory receptor neurons, they must be rapidly removed by AD and/or AOX to restore the sensitivity of the sensory neuron^16^. The *OBPs* and *CSPs* that are mainly expressed in antennae and PGs could play important roles in the binding and transport of plant volatiles, oviposition-deterring pheromones, and sex pheromones. On the other hand, antennae and PGs highly expressed *ADs*, and *AOXs*, which could be involved in degrading sex pheromone and aldehyde odorants^16^,^40^.

In conclusion, we sequenced the PG transcriptomes in the two noctuid sibling species, *H. armigera* and *H. assulta* to identify the mechanisms regulating the opposite ratios of the sex pheromone components, Z9-16:Ald and Z11-16:Ald in the two species. Our analyses based on phylogeny, transcript expression, and transcript abundance indicates that a number of transcripts with PG-biased expression could be involved in the sex pheromone biosynthesis in the two species. Particularly, *DESs* seem to play a prominent role in the regulation of the component ratio in *H. armigera* and *H. assulta.* Additional functional analyses are needed to verify this conjecture in future.

## Methods

### Insect samples

*Helicoverpa armigera* were collected from cotton fields in the Institute of Cotton Research at the Chinese Academy of Agricultural Sciences. *Helicoverpa assulta* were provided by the Henan University of Science and Technology in China. Both species were reared in the laboratory on an artificial diet^41^ at 25 ± 1°C, 14:10 L:D light cycle, and 65 ± 5% relative humidity. Pupae were sexed and kept separately in cages for eclosion. The pupae were checked daily for emergence and supplied with 10% honey solution as food for the emerging adults.

### Tissue collection

To construct cDNA libraries, 15 PGs from 3-day-old virgin females from each of the two species were collected at 5 h in scotophase (Fig. 1), immediately frozen in liquid nitrogen, and stored at −80°C until further use. In addition, for semi-quantitative RT-PCR and qRT-PCR, female antennae (FA), male antennae (MA), pheromone glands (PGs), whole insect body without pheromone glands (B1), and whole insect body without pheromone glands and antennae (B2) were also collected from three-day-old virgin insects. These tissues were immediately frozen and stored at −80°C until RNA isolation.

### cDNA library construction and Illumina sequencing

Total RNA was extracted from the PGs of *H. armigera* and *H. assulta* using TRIzol reagent (Invitrogen, Carlsbad, CA, USA), and cDNA library construction and Illumina sequencing of the samples were performed at the Beijing Genomics Institute, Shenzhen, China^42^. For each species, poly-adenylated RNA were isolated from 20 μg of pooled total RNA using oligo (dT) magnetic beads. Then, the mRNA from each species were fragmented into short pieces in the presence of divalent cations in fragmentation buffer at 94°C for 5 min. Using the cleaved fragments as templates, random hexamer primers were used to synthesize first-strand cDNA using the. Second-strand cDNA was generated using the buffer, dNTPs, RNAseH, and DNA polymerase I. Following end repair and adaptor ligation, short sequences were amplified by PCR and purified with a QIAquick® PCR extraction kit (Qiagen, Venlo, The Netherlands), and sequenced on a HisSeq™ 2000 platform (Illumina, San Diego, CA, USA). The clean reads obtained in this study are available at the NCBI/SRA database under accession numbers SRR1565435 and SRR1570898.

### Assembly and annotation

The PG transcriptomes of *H. armigera* and *H. assulta* were assembled *de novo* using the short-read assembly program Trinity^43^, which generated two classes of transcripts: clusters (prefix CL) and singletons (prefix U). Transcripts larger than 150 bp were compared to existing sequences in the protein databases, including NCBI Nr, Swiss-Prot, KEGG^44^, and COG, using blastX. We then used the Blast2GO program^45^ for GO annotation of the transcripts and WEGO software^46^ to plot the GO annotation results.

### Analysis of transcript expression in the pheromone glands

Transcript expression abundances were calculated by the RPKM (reads per kilobase per million mapped reads) method^47^, which can eliminate the influence of different transcript lengths and sequencing discrepancies in calculating expression abundance^47^. RPKM was calculated from the equation (1):
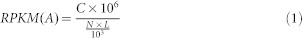
where RPKM (A) is the expression of transcript *A*; *C* is the number of reads uniquely aligned to transcript *A*; *N* is the total number of fragments uniquely aligned to all transcripts; and *L* is the number of bases in transcript *A*.

### Phylogenetic analysis

To investigate the phylogenetic relationships between the two *Helicoverpa* species, we compared all putative transcripts involved in the pheromone biosynthesis, reception, and degradation in each of the two species using ClustalX2.0 with default settings^48^. Since desaturases are the most extensively studied class of enzymes involved in sex pheromone biosynthesis, we imported 67 lepidopteran desaturases^28^ sequences from NCBI Nr and those from *H. armigera* and *H. assulta*. All phylogenetic trees were constructed using the neighbour-joining method implemented in MEGA6 with default settings and 1000 bootstrap replicates.

### Semi-quantitative RT–PCR analysis

Total RNA was isolated using the SV Total Isolation System (Promega, Madison, WI, USA) according to the manufacturer's instructions. Single-stranded cDNA templates were synthesized using 1 μg of total RNA from various samples (FA, MA, PGs, B1 and B2) using the Reverse Transcription System (Promega) following the instructions in the manual.

Specific primers for the transcripts putatively involved in pheromone biosynthesis, reception, and degradation were designed using Beacon Designer 7.7 (Premier Biosoft, Palo Alto, CA, USA) (Supplementary Table S6 online). Semi-quantitative PCR experiments with negative controls (no cDNA template) were performed as follows: 94°C for 2 min; followed by 28 cycles at 94°C for 30 sec, 60°C for 30 sec, and 72°C for 30 sec. The reactions were performed in 20 μL PCR reactions containing 2.0 μL of 10× Ex-Taq PCR buffer, 1.6 μL of dNTPs (10 mM), 0.8 μL of forward primer (10 μM), 0.8 μL of reverse primer (10 μM), 15 ng of single-stranded cDNA, and 0.2 μL Ex-Taq (5 U/μL). PCR products were analysed by electrophoresis on 2.0% w/v agarose gel in TAE buffer and the resulting bands were visualized with GluRed according to the manufacturer's instructions. The GTP-binding protein (GenBank No. AY957405) from *H. armigera* was used as an endogenous control. Each reaction had three independent biological replicates.

### Quantitative real time PCR and data analysis

Total RNA and cDNA synthesis were performed as described for semi-quantitative RT-PCR analysis. qRT-PCR was performed in a Mastercycler® ep realplex (Eppendorf, Hamburg, Germany) with primers designed based on the *Helicoverpa* nucleotide sequences from the Illumina data using Beacon Designer 7.7 (Supplementary Table S7 online). The *H. armigera* GTP-binding protein (AY957405) and GAPDH (JF417983) were used as reference genes. Expression levels of the tested mRNA were determined using the GoTaq® qPCR Master Mix (Promega) according to the manufacturer's instructions. A blank control without template cDNA (replacing cDNA with H_2_O) served as the negative control. Each reaction had three independent biological replicates and was repeated three times (technical replicates). Relative expression levels were calculated using the comparative 2^−δδCT^ method^49^.

### Statistical Analysis of data

Data (mean ± SE) from various samples were subjected to one-way nested analysis of variance (ANOVA) followed by a least significant difference test (LSD) for mean comparison. Two-sample analysis was performed by Student's *t*-test using SPSS Statistics 17.0 (IBM, Chicago, IL, USA).

## Author Contributions

J.C. and S.Z. conceived and designed the experiments; Z.L. performed the experiments; Z.L., S.D., J.L., L.L. and C.W. analysed the data; and Z.L. wrote the manuscript. All authors reviewed the final manuscript.

## Supplementary Material

Supplementary Information Supplementary Dataset 1

Supplementary Information Supplementary Dataset 2

Supplementary Information Supplementary Dataset 3

Supplementary Information Supplementary Dataset 4

Supplementary Information Supplementary Dataset 5

Supplementary Information Supplementary Dataset 6

Supplementary Information Supplementary Dataset 7

## Figures and Tables

**Table 1:** BLASTX results for candidate sex pheromone biosynthesis transcripts in *Helicoverpa armigera* pheromone glands

Transcript				Best Blastp Match				
Name	ID	ORF	RPKM	Name	Species	E-value	Identity	Acc. number
***Acetyl*-*CoA carboxylase (ACC)***								
**ACC1**	CL1009-1	1356	0.4	acetyl-CoA carboxylase-like	*Bombyx mori*	0E+00	88%	XP_004930758
**ACC2**	CL1295-1	5211	40.9	acetyl-coA carboxylase	*Agrotis ipsilon*	0E+00	95%	AGR49308
***Aldo-Ketose Reductase (ALR)***								
**ALR1**	CL2516-1	1077	10.5	alcohol dehydrogenase	*Aedes aegypti*	1E-173	67%	XP_001655101
**ALR2**	CL3786-1	420	323.6	alcohol dehydrogenase	*Bombyx mori*	1E-38	57%	XP_004922743
**ALR3**	CL4692-1	918	23.2	alcohol dehydrogenase	*Bombyx mori*	5E-165	71%	XP_004922743
**ALR4**	CL5008-1	312	8.9	alcohol dehydrogenase, partial	*Agrotis ipsilon*	5E-26	55%	AGQ45607
**ALR5**	CL5271-5	1002	41.2	alcohol dehydrogenase, partial	*Agrotis ipsilon*	1E-73	52%	AGQ45607
**ALR6**	CL5277-1	682	112.4	alcohol dehydrogenase	*Danaus plexippus*	3E-102	66%	EHJ65258
**ALR7**	CL5878-1	306	8.8	alcohol dehydrogenase, partial	*Agrotis ipsilon*	5E-50	79%	AGQ45610
**ALR8**	CL6326-1	360	9.9	putative alcohol dehydrogenase	*Danaus plexippus*	3E-48	68%	EHJ73729.1
**ALR9**	U10235	426	3.1	putative alcohol dehydrogenase	*Danaus plexippus*	6E-81	84%	EHJ71310.1
**ALR10**	U11986	306	3.3	putative alcohol dehydrogenase	*Danaus plexippus*	2E-10	41%	EHJ68420
**ALR11**	U12541	231	6.3	alcohol dehydrogenase, partial	*Agrotis ipsilon*	2E-19	59%	AGQ45607.1
**ALR12**	U13468	289	2.8	putative alcohol dehydrogenase	*Danaus plexippus*	2E-37	73%	EHJ68420.1
**ALR13**	U13469	358	7.2	putative alcohol dehydrogenase	*Danaus plexippus*	7E-27	60%	EHJ68420.1
**ALR14**	U17782	663	10.9	putative alcohol dehydrogenase	*Danaus plexippus*	1E-130	79%	EHJ73729.1
**ALR15**	U19886	975	34.4	alcohol dehydrogenase	*Bombyx mori*	0E+00	78%	XP_004921850.1
**ALR16**	U21480	750	11.4	alcohol dehydrogenase, partial	*Agrotis ipsilon*	8E-81	52%	AGQ45608.1
**ALR17**	U21731	1131	52.5	alcohol dehydrogenase	*Bombyx mori*	0E+00	95%	NP_001040507.1
***Desaturase (DES)***								
**KPSE**	CL1090-3	1062	16.6	acyl-CoA delta-9 desaturase	*Helicoverpa zea*	2E-171	100%	AAF81788.1
**NPVE**	CL1090-4	1062	4364.0	acyl-CoA delta-9 desaturase	*Helicoverpa zea*	0E+00	100%	AAF81790.2
**MPVE**	CL1931-1	900	13.4	acyl-CoA Delta(11) desaturase	*Bombyx mori*	2E-09	72%	XP_004925564.1
**GATD**	U23856	1119	41.5	acyl-CoA desaturase HassGATD	*Helicoverpa assulta*	0E+00	98%	AAM28480.2
**LPAQ**	U23789	1017	3975.6	acyl-CoA delta-11 desaturase	*Helicoverpa zea*	0E+00	99%	AAF81787.1
**KSVE**	U21458	1119	64.0	acyl-CoA desaturase HvirKSVE	*Heliothis virescens*	0E+00	98%	AGO45842.1
**NRPE**	U27960	822	3.5	acyl-CoA Delta(11) desaturase	*Bombyx mori*	0E+00	92%	XP_004932163.1
***Fatty acid synthase (FAS)***								
**FAS1**	CL2920-1	3843	4.3	fatty acid synthase	*Agrotis ipsilon*	0E+00	92%	AGR49310.1
**FAS2**	U17719	2798	237.5	fatty acid synthase	*Agrotis segetum*	0E+00	92%	AID66645.1
**FAS3**	U17720	1177	65.8	fatty acid synthase	*Agrotis ipsilon*	0E+00	91%	AGR49310.1
***Fatty acyl*-*CoA reductase (FAR)***								
**FAR1**	CL1521-1	516	46.3	putative fatty acyl-CoA reductase	*Agrotis ipsilon*	8E-109	91%	AGR49318.1
**FAR2**	CL1525-1	1572	58.4	fatty-acyl CoA reductase 6, partial	*Agrotis ipsilon*	0E+00	72%	AGR49316.1
**FAR3**	CL1589-2	501	1.6	fatty acid reductase	*Helicoverpa assulta*	4E-35	38%	AFD04727.1
**FAR4**	CL1835-1	1270	17.4	fatty-acyl CoA reductase 2	*Ostrinia nubilalis*	0E+00	81%	ADI82775.1
**FAR5**	CL3768-1	1614	69.7	putative fatty acyl-CoA reductase	*Bombyx mori*	0E+00	75%	XP_004926017.1
**FAR6**	CL4218-1	366	14.4	fatty-acyl CoA reductase 6	*Agrotis ipsilon*	5E-57	89%	AGR49326.1
**FAR7**	CL4398-1	909	4.0	fatty-acyl CoA reductase 5	*Danaus plexippus*	3E-129	76%	EHJ72233.1
**FAR8**	CL5981-1	1266	45.5	fatty-acyl CoA reductase 6	*Danaus plexippus*	0E+00	64%	EHJ76493.1
**FAR9**	CL6073-1	1557	39.2	putative fatty acyl-CoA reductase	*Bombyx mori*	0E+00	81%	XP_004929961.1
**FAR10**	CL6322-1	861	87.9	putative fatty acyl-CoA reductase	*Agrotis ipsilon*	6E-175	85%	AGR49318.1
**FAR11**	CL6616-1	1424	59.9	putative fatty acyl-CoA reductase	*Bombyx mori*	0E+00	83%	XP_004925992.1
**FAR12**	CL7377-1	1371	414.2	fatty acid reductase	*Helicoverpa assulta*	0E+00	99%	AFD04727.1
**FAR13**	U2195	1497	22.0	fatty-acyl CoA reductase 4	*Ostrinia nubilalis*	0E+00	68%	ADI82777.1
**FAR14**	U24540	417	23.4	putative fatty acyl-CoA reductase	*Agrotis ipsilon*	1E-86	95%	AGR49319.1
**FAR15**	U24542	936	22.2	putative fatty acyl-CoA reductase	*Agrotis ipsilon*	0E+00	94%	AGR49319.1
**FAR16**	U25481	201	40.3	fatty-acyl CoA reductase 5	*Ostrinia nubilalis*	3E-22	63%	ADI82778.1
**FAR17**	U25568	405	22.7	fatty-acyl CoA reductase 2	*Ostrinia nubilalis*	7E-68	74%	ADI82775.1
**FAR18**	U32	564	18.1	fatty-acyl CoA reductase 5	*Danaus plexippus*	2E-94	75%	EHJ72233.1

**Table 2:** BLASTX results for putative sex pheromone biosynthesis transcripts in *Helicoverpa assulta* pheromone glands

Transcript				Best Blastp Match				
Name	ID	ORF	RPKM	Name	Species	E-value	Identity	Acc. number
***Acetyl*-*CoA carboxylase (ACC)***								
**ACC1**	U22914	510	7.7	cetyl-coA carboxylase, partial	*Agrotis ipsilon*	1E-80	79%	AGR49309.1
**ACC2**	CL1044-1	4983	41.5	acetyl-coA carboxylase	*Agrotis ipsilon*	0E+00	95%	AGR49308.1
***Aldo-Ketose Reductase (ALR)***								
**ALR1**	U4829	320	4.0	alcohol dehydrogenase	*Culex quinquefasciatus*	2E-17	86%	XP_001848848
**ALR2**	CL3549-1	483	8.8	alcohol dehydrogenase, partial	*Agrotis ipsilon*	3E-44	55%	AGQ45608.1
**ALR3**	CL3700-1	813	7.2	putative alcohol dehydrogenase	*Danaus plexippus*	3E-62	47%	EHJ68420.1
**ALR4**	U1366	198	5.7	alcohol dehydrogenase, partial	*Agrotis ipsilon*	6E-18	59%	AGQ45607.1
**ALR5**	CL2456-2	1002	10.7	alcohol dehydrogenase, partial	*Agrotis ipsilon*	0E+00	79%	AGQ45607.1
**ALR6**	U18627	753	521.2	alcohol dehydrogenase, partial	*Agrotis ipsilon*	7E-142	78%	AGQ45608.1
**ALR7**	U6810	975	14.9	putative alcohol dehydrogenase	*Danaus plexippus*	5E-133	64%	EHJ73729.1
**ALR8**	U4937	342	5.5	putative alcohol dehydrogenase	*Danaus plexippus*	2E-21	59%	EHJ68420.1
**ALR9**	U12805	1017	9.6	aldose reductase-like	*Bombyx mori*	4E-176	71%	XP_004921845
**ALR10**	U7365	305	2.9	putative alcohol dehydrogenase	*Danaus plexippus*	2E-25	54%	EHJ71310.1
**ALR11**	U8744	813	3.3	putative alcohol dehydrogenase	*Danaus plexippus*	2E-117	62%	EHJ70606.1
**ALR12**	U23789	483	3.8	putative alcohol dehydrogenase	*Bombyx mori*	1E-69	65%	NP_001037610
**ALR13**	CL115-1	759	0.0	alcohol dehydrogenase, partial	*Agrotis ipsilon*	2E-118	62%	AGQ45606.1
**ALR14**	U9712	1071	9.0	putative alcohol dehydrogenase	*Danaus plexippus*	0E+00	74%	EHJ73729.1
**ALR15**	U19322	975	66.1	alcohol dehydrogenase	*Bombyx mori*	0E+00	77%	XP_004921850
**ALR16**	U4138	694	4.2	putative alcohol dehydrogenase	*Danaus plexippus*	2E-49	82%	EHJ71310.1
**ALR17**	U1545	1131	106.3	alcohol dehydrogenase	*Bombyx mori*	0E+00	95%	NP_001040507
**ALR18**	U24329	366	3.2	putative alcohol dehydrogenase	*Bombyx mori*	7E-36	78%	NP_001037610
***Desaturase (DES)***								
**KPSE**	U18841	1062	659.9	acyl-CoA delta-9 desaturase	*Helicoverpa zea*	0E+00	100%	AAF81788.1
**NPVE**	U21938	1062	949.2	acyl-CoA desaturase HassNPVE	*Helicoverpa assulta*	0E+00	99%	AAM28484.2
**MPVE**	U1020	1104	16.8	acyl-CoA Delta(11) desaturase-like	*Bombyx mori*	1E-173	65%	XP_004925564
**GATD**	U18038	1119	40.0	acyl-CoA desaturase HassGATD	*Helicoverpa assulta*	0E+00	99%	AAM28480.2
**LPAQ**	U21077	1017	132.9	acyl-CoA desaturase HassLPAQ	*Helicoverpa assulta*	0E+00	99%	AAM28483.2
**KSVE**	U21918	1119	26.9	acyl-CoA desaturase HvirKSVE	*Heliothis virescens*	0E+00	98%	AGO45842.1
**KSPP**	U12152	892	6.5	acyl-CoA Delta(11) desaturase-like	*Bombyx mori*	1E-160	75%	NP_001274329
**TYSY**	CL2025-1	966	20.3	desaturase	*Agrotis segetum*	0E+00	94%	AID66658.1
***Fatty acid synthase (FAS)***								
**FAS1**	U13060	910	4.2	fatty acid synthase-like	*Bombyx mori*	4E-82	48%	XP_004927661
**FAS2**	U22164	7170	345.7	fatty acid synthase	*Agrotis ipsilon*	0E+00	92%	AGR49310.1
**FAS3**	U2985	271	3.7	fatty acid synthase-like	*Bombyx mori*	8E-24	73%	XP_004922804
***Fatty acyl*-*CoA reductase (FAR)***								
**FAR1**	CL3772-1	1614	7.8	putative fatty acyl-CoA reductase	*Bombyx mori*	0E+00	75%	XP_004926017
**FAR2**	U795	1497	30.4	fatty-acyl CoA reductase 4	*Ostrinia nubilalis*	0E+00	69%	ADI82777.1
**FAR3**	U1030	1488	101.1	putative fatty acyl-CoA reductase	*Agrotis ipsilon*	0E+00	94%	AGR49319.1
**FAR4**	U1584	1575	55.6	fatty-acyl CoA reductase 6	*Danaus plexippus*	0E+00	63%	EHJ76493.1
**FAR5**	U18296	1557	26.0	putative fatty acyl-CoA reductase	*Bombyx mori*	0E+00	80%	XP_004929961
**FAR6**	U20971	1371	960.1	fatty acid reductase	*Helicoverpa assulta*	6E-108	100%	AFD04727.1
**FAR7**	U22269	1569	94.4	fatty-acyl CoA reductase 3	*Ostrinia nubilalis*	0E+00	80%	ADI82776.1
**FAR8**	CL283-1	1533	14.7	putative fatty acyl-CoA reductase	*Agrotis ipsilon*	0E+00	87%	AGR49318.1
**FAR9**	U25153	244	2.7	putative fatty acyl-CoA reductase	*Bombyx mori*	1E-25	64%	XP_004925987
**FAR10**	U25265	305	2.0	putative fatty acyl-CoA reductase	*Bombyx mori*	2E-51	91%	XP_004930776
**FAR11**	CL598-1	1572	0.3	fatty-acyl CoA reductase 6, partial	*Agrotis ipsilon*	0E+00	71%	AGR49316.1
**FAR12**	CL1250-1	1617	0.1	fatty-acyl CoA reductase 5	*Danaus plexippus*	0E+00	74%	EHJ72233.1
**FAR13**	CL1309-1	488	16.7	fatty-acyl CoA reductase 2	*Ostrinia nubilalis*	0E+00	81%	ADI82775.1
